# The promise of automated machine learning for the genetic analysis of complex traits

**DOI:** 10.1007/s00439-021-02393-x

**Published:** 2021-10-28

**Authors:** Elisabetta Manduchi, Joseph D. Romano, Jason H. Moore

**Affiliations:** 1 Department of Biostatistics, Epidemiology and Informatics, University of Pennsylvania, Philadelphia, PA 19104 USA; 2 Institute for Biomedical Informatics, University of Pennsylvania, Philadelphia, PA 19104 USA

## Abstract

The genetic analysis of complex traits has been dominated by parametric statistical methods due to their theoretical properties, ease of use, computational efficiency, and intuitive interpretation. However, there are likely to be patterns arising from complex genetic architectures which are more easily detected and modeled using machine learning methods. Unfortunately, selecting the right machine learning algorithm and tuning its hyperparameters can be daunting for experts and non-experts alike. The goal of automated machine learning (AutoML) is to let a computer algorithm identify the right algorithms and hyperparameters thus taking the guesswork out of the optimization process. We review the promises and challenges of AutoML for the genetic analysis of complex traits and give an overview of several approaches and some example applications to omics data. It is our hope that this review will motivate studies to develop and evaluate novel AutoML methods and software in the genetics and genomics space. The promise of AutoML is to enable anyone, regardless of training or expertise, to apply machine learning as part of their genetic analysis strategy.

## Outline

We provide a review of current Automated Machine Learning (AutoML) systems with a special emphasis on their applicability to the biomedical domain and, in particular, on omics and genetic applications. The content is organized as follows. In the next section, we describe Machine Learning (ML) in general, including open-source ML tools, feature importance, and biomedical applications. In the following section, we focus on AutoML, describing first three widely used open-source solutions (Auto-WEKA, Auto-sklearn, and TPOT). Then, we present AutoML solutions that include neural networks or allow for neural architecture search. Finally, we briefly discuss a few additional systems, including commercial ones. The next section focuses on TPOT applications to omics, since this tool has been particularly used in this context and some of its refinements were motivated by this type of applications. We conclude in the next section with a discussion of promises and challenges of AutoML in the genetics domain, including the typically large dimensionality of genetics data and class imbalance.

## Machine learning

### Generalities

Machine Learning (ML) refers to approaches by which computers learn from data to accomplish certain tasks, without a programmer having to specify every single algorithmic instruction. In supervised ML, which will be the focus of what follows, the task is to learn a predictive model from *training data* that provide examples of inputs and their corresponding outputs. This means learning a general rule that can then be used to predict the outputs for new inputs of the same type as the training data. Each input consists of the values that a collection of *features* (the independent/explanatory variables, also referred to as *predictors*) have for a particular sample (e.g. an individual or subject, also referred to as *observation*), and the output is the value of a *target outcome* of interest (the dependent/response variable) for that sample. Inputs can be represented by a matrix *X* whose columns correspond to *p* features and rows to *n* samples and the output by a vector *y* of *n* target values for those samples. In a *classification* problem the target can take finitely many possible values (class labels), whereas in a *regression* problem, the target is continuous. Any given algorithm in ML can have *parameters* and *hyperparameters*. Parameters are internal configuration variables learned from the data during training. For example, the coefficients in a linear or logistic regression model are parameters for that model. Hyperparameters are instead values that are specified before the learning starts. For example, in regularized regression, a penalty hyperparameter λ is specified to discourage complex models; in a random forest (RF), the number of trees is a hyperparameter, etc.

**Fig. 1:**
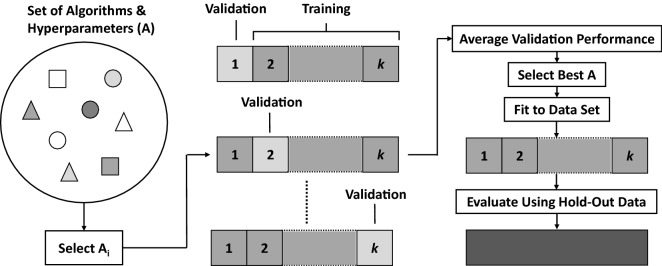
Flow for *k*-fold CV on algorithm/hyperparameter selection and evaluation. *A*_*i*_ indicates the selection of an algorithm with specified hyperparameters

There are several metrics that can be used to assess the effectiveness of a predictive model. In classification problems, a frequently used metric is the accuracy (i.e., the percentage of correct predictions) or its variants which are appropriate in the presence of imbalance between the number of samples from the different classes, such as the balanced accuracy. Metrics frequently used for regression problems include the root mean squared error (RMSE), i.e. the square root of the average squared difference between true and predicted values, and the coefficient of determination, which reflects the proportion of target variance explained by the model. But there are many other choices of metrics that can be selected when optimizing algorithms and hyperparameters in an ML application (Kuhn and Johnson 2013; Zheng 2015). An ML model’s performance should be evaluated by computing its score for the chosen metric on a data set separate from that used for training. The score on the training set is not a good indicator of the generalizability of the learned model as it could be affected by *overfitting*, a phenomenon by which the model has adapted to characteristics which are specific to the training set. Thus, the typical flow is to use a training set to learn the model parameters, i.e. to *fit* the model, and then to assess the model performance on a hold-out *testing set*, with samples drawn from the same population. Often, it is desirable to tune the choice of algorithm and its hyperparameters. This can be done using an independent *validation set*, with samples drawn from the same population as the training set. Essentially, for each of different choices of algorithm and of its hyperparameter settings, one fits the model with those selections to the training set and then evaluates its performance on the validation set. The model with the choices that optimize performance on the validation set is then selected and evaluated on the hold-out testing set. In practice, more complex schemes are employed. For example, a common approach is *k*-fold cross-validation (CV), where the input data are randomly partitioned into *k* equal sized subsets. For each of different choices of algorithm and of its hyperparameter settings, in turn one of the subsets serves as the validation set and the model is fit to the union of the remaining *k *– 1 subsets and evaluated on the validation set. Then, the selection yielding the best average performance across the *k* folds is adopted and the corresponding model is fit to the entire training set and evaluated on a hold-out testing set (see Fig. 1).

There are several steps involved in setting up a full ML solution for a given task, from pre-processing to predictive model generation. First, the data must be cleaned as appropriate; this includes decisions on how to handle missing values, how to encode nominal variables, etc. Then, prior to running a classifier or regressor ML algorithm, one needs to decide whether to use all or a subset of the features and, in the latter case, what algorithm to use to select such features. Moreover, one needs to decide on possible transformations to apply to the features and creation of new features, which is referred to as *feature engineering*. Thus, in effect, the typical solution consists of a pipeline of feature selector, feature transformation, and estimator algorithms (classifiers or regressors), where the output of a step becomes the input of the following step. Each pipeline could involve more than one feature selector, feature transformer, and estimator. Indeed, multiple pipelines could be combined in a workflow, yielding an even more complex architecture. The ultimate goal is to obtain a solution with good predictive performance, but at the same time there is a tradeoff between complexity and interpretability; a simpler solution may be preferable to a very complex one if the latter is only slightly better in terms of performance. Thus, many decisions must be made, different options assessed (including hyperparameter settings), and in general, the process is labor intensive and requires considerable domain expertise.

### Open-source software

Along with scientific software used in other domains, ML has benefitted substantially from the “free and open-source software” (FOSS) movement. “Free” software (or libre software) is computer software that can be used for any purpose without restrictions, including modifying and/or redistributing the software. “Open-source” specifically refers to making the source code for the software publicly available, either by distributing the software directly as source code at no cost, or by maintaining a separate source code repository (e.g., GitHub, Sourceforge, or similar) that the public can use to browse the source code directly through a web browser or other interfaces. Most popular ML software today is open-source, and even many ML frameworks developed by large corporations are released as open-source and developed publicly, often allowing for external contributions and improvements from end-users.

WEKA (Frank et al. 2016) is one of the earliest open-source ML software still in common use, originally developed in 1993 at the University of Waikato. WEKA supports classification, regression, pre-processing, and other common data mining tasks, and provides a graphical user interface catered towards users with little to no programming experience or users who prefer not to work in a command line environment. Deep learning (described below) is supported via the Deeplearning4j library for the Java programming language.

Scikit-learn (Pedregosa et al. 2011) is one of the most popular ML libraries and acting as one of the main interfaces for ML in the Python programming language. Scikit-learn focuses on providing a common, easy-to-use application programming interface (API) for a wide range of ML tasks, and supports advanced features including pipeline construction, semi-supervised learning, and others. Many other ML toolkits imitate or extend the scikit-learn API due to its familiarity and simplicity.

Several open-source programming languages have ML features implemented either as part of the core language or in the language’s standard library. The R programming language—designed mainly for statistical computing—implements a number of ML algorithms as core functions that are actively loaded at all times. For example, the *k*-nearest neighbors algorithm can be trained on a dataset by calling knn(*X*_train, *X*_test, *y*_train), where *X* and *y* are features and targets (class labels), respectively, and ‘train’ and ‘test’ refer to training and testing datasets, respectively (note that no import statements or other external libraries are needed). The Julia programming language offers similar basic functionality as part of the language’s standard library, but most Julia users apply ML algorithms using the MLJ.jl library, developed and released as free and open-source by the Alan Turing Institute.

### Feature importance

After a predictive model has been built, it is usually of interest to explore which features are driving the model. There are various approaches to rank features in terms of their importance for the model. Some estimators naturally yield quantities that can serve this purpose. For example, the coefficients of a linear or logistic regression model reflect feature importance when the features have the same scale; in decision trees (and their ensembles, such as RFs), the criteria used to select the split points yield importance scores. A general method which can be employed with any estimator (i.e., *model-agnostic*) is *permutation importance* (see https://github.com/TeamHG-Memex/eli5 for an implementation). The idea is to measure feature importance for each feature by examining how much the selected performance score (e.g., accuracy, or RMSE, etc.) degrades when that feature is not available. However, removing one feature at a time, retraining the model, and computing the new score would be too intensive computationally. Instead, after the model is fit, for each feature, its values in the hold-out testing set are permuted and the model evaluated on the resulting set (typically multiple permutations are applied and the resulting scores averaged). Essentially, instead of removing that feature, one replaces it by random noise. Features can then be ranked according to how much worse performance on the permuted set is as compared to performance on the unpermuted set. We note that this approach should be used with care, as discussed in Molnar et al. (2021). For example, if features are correlated, permuting a feature could produce unrealistic data instances which could lead to misleading results. The effects of breaking feature dependencies in various model setups are explored in Hooker et al. (2021) and alternative approaches are discussed, which, however, require more computation. Another point to keep in mind to avoid misleading interpretations, is that permutation importance does not separate between main and interaction effects, but reflects both the importance of a feature as well as that of its interactions with the other features (Casalicchio et al. 2019; Molnar et al. 2021).

Permutation importance measures the overall relevance of a feature to a model; it is a so-called ‘global’ method. However, it is also of interest to examine how each feature contributes to the individual predictions. This is particularly relevant in the context of precision medicine and when there is heterogeneity among subjects (i.e., when different features are responsible for the same outcome in different subjects). Approaches have recently been developed with this goal in mind; these are termed ‘local’ methods. For example, SHAP (Lundberg and Lee 2017) are game-theory-derived metrics indicating, for each sample, how much each feature contributed to the model prediction for that sample. These values can also be summarized across samples to rank features according to their overall contribution to all predictions. SHAP values need to be used with care too. For example, KernelSHAP, a model-agnostic method to compute them, ignores feature dependencies. TreeSHAP, a method to compute SHAP values for tree-based models, does not have this problem but could assign nonzero values to features that have no influence on the prediction.

Feature importance falls within the umbrella of interpretability in ML, a relevant and complex research area. There are other model-agnostic global and local methods besides those mentioned above. An overview of these and a guide to interpretable ML in general is provided by Molnar (2021).

### Biomedical applications

ML approaches are now routinely used in biomedical applications, as an alternative or a complement to statistical approaches. This includes applications leveraging omics data. For example, Bazaga et al. (2020) utilize multiple ML algorithms to build drug-target prediction models for a variety of cancer types. The features include genetic mutations, gene expression, gene essentiality, and gene interactions. The resulting models are then applied to more than 15,000 protein coding genes to identify novel cancer type-specific drug-target candidates. In Adams et al. (2020), an RF-driven method is applied to a genome-wide association study (GWAS) to identify epistasis-networks that may provide insights into the risk for on-statin major adverse cardiovascular events. More generally, RFs have been an effective ML approach to identify epistasis, i.e. interactions between two or more genetic loci which are associated to a given phenotype (Orlenko and Moore 2021). Another type of ML application specific to genomics consists of building classifiers for deleterious versus non-deleterious genetic variants. For example, CADD (Rentzsch et al. 2019) is based on a logistic regression model trained on more than 30 million variants and leveraging features from 60 different annotations, including conservation, epigenetic modifications, functional predictions, and genetic context. Using this model CADD then generated deleteriousness scores for variants throughout the human genome reference assembly. Another example is GWAVA (Ritchie et al. 2014), which employs RFs trained on functional genomics features to build a variant prioritization tool for non-coding variants. RFs are also used in TraP (Gelfman et al. 2017) to build an annotator for pathogenic non-coding variants in genic regions, and in REVEL (Ioannidis et al. 2016), to predict the pathogenicity of rare missense variants.

One important consideration for ML applications to omics data is the ‘big *p*, little *n*’ (*p* >  > *n*) issue. In the omics context, unlike the more traditional tabular data to which ML is often applied, the number *p* of predictors is usually much larger than the number of observations. This *curse of dimensionality* makes it challenging to have a sufficiently representative sample of the *p*-dimensional feature domain, needed to build a good predictive model which does not suffer from overfitting. Thus, applications of ML to omics typically require pre-processing steps to reduce the number of predictors in the input to the ML. These include feature selection, based on expert knowledge or computationally based, and feature transformations aimed at dimensionality reduction.

## Automated machine learning

According to the No Free Lunch Theorems (Wolpert 1996; Wolpert and Macready 1997), there is no single ML algorithm that works well on all tasks. Every aspect of an ML application needs careful configuration and, as we have indicated above, setting up a pipeline requires considerable ML experience to best tune it for the specific task at hand. Therefore, methods which can assist in the design and optimization of ML pipelines, referred to as Automated Machine Learning (AutoML), are particularly appealing to biomedical investigators with limited data science expertise. There are approaches aimed at automating single tasks of an ML pipeline, such as feature engineering or hyperparameter optimization for a specified algorithm, but AutoML methods that can handle multiple tasks are particularly appealing as they provide off-the-shelf options for non-expert users. Below, we discuss several AutoML approaches, summarized in Table 1. For an in-depth description of fundamentals and an extensive review of state-of-the-art AutoML methods, we refer the reader to Hutter et al. (2019) and, with a healthcare perspective, to Waring et al. (2020).

**Table 1:** For each of the AutoML systems that we discussed, the architecture type of the resulting pipeline and optimization method are indicated together with the type of applications described in this work

System	Pipeline architecture	Optimization method	Application type
AutoGluon	Layers	Stacked Ensenble	B
AutoPrognosis	Ensemble	BO	B
Auto-sklearn	Fixed	CASH via BO (SMAC)	B
Auto-WEKA	Fixed (and simple)	CASH via BO (SMAC)	B
H2O	Ensemble	BO and SuperLearner	
Model search	NN	NAS via GP	
PennAI	Fixed (and simple)	Recommender	B
TPOT	Flexible	CASH via GP	B, O, G
TPOT-NN	NN	GP	

*BO* Bayesian optimization, *B* biomedical, not omics, *O* omics, but not genomics, *G* genomics

### Open-source AutoML pipeline optimization methods

Here, we adopt the terminology by Waring et al. (2020) and refer to pipeline optimization AutoML as those methods which address more than one task in an ML pipeline. The three most popular open-source pipeline optimization AutoML systems to date are Auto-WEKA (Thornton et al. 2013; Kotthoff et al. 2019), built on top of the WEKA package; Auto-sklearn (Feurer et al. 2015), built on top of the scikit-learn package; and Tree-based Pipeline Optimization Tool (TPOT) (Olson et al. 2016; Olson and Moore 2019), which also leverages scikit-learn. All three of these approaches aim to solve the Combined Algorithm Selection and Hyperparameter (CASH) optimization problem. The idea is to also model the choices of algorithms for pipeline steps as hyperparameters and then consider conditional dependencies between hyperparameters, i.e., a hyperparameter may be relevant only when other hyperparameters have certain values. For example, if the hyperparameter ‘estimator algorithm’ takes the value ‘random forest’, then the RF hyperparameters become relevant. Thus, the entire pipeline optimization task can be formulated in terms of a structured hyperparameter optimization problem. Auto-WEKA and Auto-sklearn optimize pipelines with a fixed architecture, in terms of the number and type of pipeline steps, whereas TPOT supports arbitrarily sized and complex pipelines by leveraging genetic programming as we illustrate below. Both Auto-WEKA and Auto-sklearn are based on Bayesian optimization (Brochu et al. 2010). Bayesian optimization aims to find the optimal architecture quickly without reaching a premature sub-optimal architecture, by trading off exploration of new (hence high-uncertainty) regions of the search space with exploitation of known good regions. This is achieved by generating a probabilistic model capturing the relationship between hyperparameter settings and performance, using this model to select the next most promising hyperparameter setting, updating the model with the result from evaluation at the new setting, and iterating.

#### Auto-WEKA

Auto-WEKA is an Auto ML extension built on top of WEKA (discussed above), designed primarily for users without the technical expertise to know which particular ML algorithm or hyperparameter settings are ideal for the specific task they are performing. Auto-WEKA is a level of abstraction that treats the entirety of WEKA as a single ML algorithm comprised of other, specific ML algorithms:

$$\mathcal{A}=\{{A}^{\left(1\right)},\dots ,{A}^{\left(k\right)}\}.$$

Briefly, the goal of Auto-WEKA is to find the correct algorithm ${A}^{\left(i\right)}\in \mathcal{A}$—and the correct hyperparameter settings for that algorithm—resulting in the best CV performance on a user-provided training data set. This is done using the previously mentioned Bayesian optimization approach for the CASH problem. A Bayesian approach provides the flexibility for specific applications to choose their own optimization model, but an effective one should be able to handle the tradeoff between model complexity and computational performance, while simultaneously choosing sensible prior distributions and initial parameterizations. The specific Bayesian optimization algorithm used in Auto-WEKA is Sequential Model-based Algorithm Configuration (SMAC), which is one of several evaluated during the project’s original development. From a high level, SMAC iteratively selects candidate algorithms for evaluation and uses both prior knowledge (based on similar problems) and results from previous iterations to propose new sets of hyperparameters for them that are likely to perform well on the learning task. At the beginning of an experiment, SMAC evaluates all candidate algorithms to identify their best initial parameter set ${\varvec{\lambda}}$ based on their *expected positive improvement*, which is computed by optimizing the expectation of a loss function defined over the means and standard deviations of the parameters in ${\varvec{\lambda}}$. At each iteration, this value is maximized based on the current model, the models are evaluated using these new optimal parameters, the average CV loss of those parameters on a training data set is computed, and the model is updated based on the actual improved value. Specifically, this expectation is defined over ${M}_{L}$—a predictive model for the best algorithm along with its optimal hyperparameter configuration, which is implemented in SMAC as a random forest.

In the case of Auto-WEKA, the candidate algorithms consist of 39 (as of Auto-WEKA 2.0’s initial release) algorithms contained within WEKA, each of which falls into one of 4 categories: *learners*, *ensemble methods*, *meta-methods*, and *attribute selection methods* (i.e., feature selectors). A complete list of the candidate algorithms is given in Kotthoff et al. (2017). “Prior knowledge” consists of a database of candidate algorithms and hyperparameter configurations previously evaluated within the same run of Auto-WEKA, and accordingly the estimation capacity of SMAC improves at each iteration of the algorithm’s main loop. At the beginning of an Auto-WEKA experiment, all parameters for all algorithms are assigned either a uniform or log-uniform prior (as semantically appropriate for each algorithm). Comprehensive details on SMAC and how it is used in conjunction with Auto-WEKA are given in Hutter et al. (2011) and Kotthoff et al. (2019). When run on a set of training data, the output of Auto-WEKA is a trained model comprised of a specific ML algorithm with hyperparameters identified by SMAC. For example, this might consist of an RF for the algorithm and certain values for number of iterations, minimum number of instances per leaf, maximum depth of trees, and others as the optimized hyperparameters (these are specific to RF, and would change if a different ML algorithm was selected). In contrast, competing AutoML software implementations (such as Auto-sklearn and TPOT; see below) often produce pipelines comprising multiple steps (data preprocessors, estimators, and meta-operators) that manipulate the data in various ways that are often crucial in real-world applications of ML.

Biomedical applications of Auto-WEKA currently in the literature include using genotypes and biochemical laboratory values to predict liver fibrosis in patients with hepatitis C (Shousha et al. 2018), predicting functional outcome scores for brain hemorrhage patients using combined demographic, laboratory, and radiometric imaging data (Wang et al. 2019), and various applications in quantitative structure/activity relationship (QSAR) modeling (Nantasenamat et al. 2015).

#### Auto-sklearn

Auto-sklearn, like Auto-WEKA, tackles the CASH problem using the SMAC Bayesian optimization method, but it combines it with an initial warm starting step to improve efficiency and a final (optional) ensemble step to improve performance and reduce overfitting. The warm starting step consists of initializing the Bayesian optimizer with hyperparameter settings based on meta-learning. More precisely, an a priori step, performed just once by the Auto-sklearn maintainers, uses Bayesian optimization to determine optimal settings for a large number of data sets in the OpenML repository (Vanschoren et al. 2014). Each data set in this repository is summarized by a set of meta-features, such as the number of data points, features, and classes, the data skewness, the entropy of the targets, etc. When Auto-sklearn is run on a new dataset, its meta-features are computed and the precomputed hyperparameter settings for the (25) most similar (based on meta-features) data sets in the repository are used to initialize the optimizer. Once warm started, the optimizer searches trough pipelines whose architecture consists of zero or one *feature preprocessors* (feature selectors or transformers which change the set of input features), up to three *data preprocessors* (transformers which change the feature values) and an estimator. There are several possible choices for the algorithms for each of these steps drawn from scikit-learn. At the end of optimization an optional post hoc step allows the user to request, instead of the best pipeline from the optimizer, an ensemble of the pipelines stored during optimization constructed using a method described in Caruana et al. (2004). When running Auto-sklearn, a user specifies the resource limits (memory and time) which is necessary, especially when working with large data sets. Of course, there is a tradeoff between resource limits and number of pipelines that can be tested.

Auto-sklearn has been applied very successfully to data sets from the ChaLearn AutoML challenges (Guyon et al. 2019), winning in several phases of these challenges. A search on PubMed (http://pubmed.ncbi.nlm.nih.gov) has yielded a few works employing Auto-sklearn in the biomedical context (Padmanabhan et al. 2019; Howard et al. 2020; Tran et al. 2020). We have not identified in the literature any Auto-sklearn application to omics data, likely due to the challenging size of these types of data sets. As progress is being made towards handling large data sets more effectively, such as in the recent extension PoSH Auto-sklearn (Feurer et al. 2018), we expect to see the use of this system also in the omics field.

#### TPOT

**Fig. 2:**
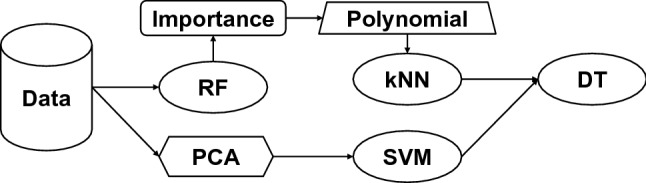
A hypothetical machine learning pipeline which could be discovered by TPOT. In the top branch of the pipeline, features are selected from a random forest (RF) analysis according to their importance scores and then subjected to a polynomial transformation. The transformed features are then analyzed using a *k*-nearest neighbors (kNN) algorithm with the output given to a decision tree (DT) as a new engineered feature. In the bottom branch, principal components (PCA) are analyzed by a support vector machine (SVM) with the output given to the DT. The DT performs the final classification using the newly engineered features from the kNN and SVM

Whereas Auto-WEKA and Auto-sklearn optimize pipelines with a fixed architecture, TPOT allows for arbitrarily sized ML pipelines. These pipelines involve operators (e.g. feature selectors, feature transformers, and ML estimators) drawn from scikit-learn and XGBoost (Chen and Guestrin 2016), as illustrated by the example in Fig. 2.

**Fig. 3:**
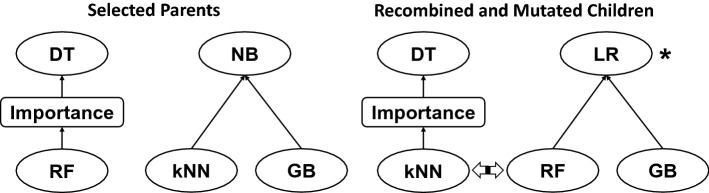
The essence of genetic programming-based optimization is the selection of good AutoML pipelines (parents) and the introduction of variability to generate new pipelines (children) for evaluation. On the left are two selected parental pipelines. In the first pipeline, features are selected according to their importance scores from a random forest (RF) analysis and then given to a decision tree (DT) which performs the classification. The second pipeline performs a k-nearest neighbors (kNN) and gradient boosting (GB) analysis with the output given to a naïve Bayes (NB) algorithm for classification. Two new pipelines are created by randomly swapping or recombining the RF and kNN algorithms and mutating the NB algorithm to a logistic regression (LR) algorithm. This results in two new pipelines to be evaluated

TPOT tackles the CASH problem using genetic programming (GP). It starts by generating an initial population of *N* random pipelines (the population size *N* defaults to 100 but can be user-specified) and evaluates them using the average *k*-fold CV score on the input data set (*k* defaults to 5 but can be user-specified), where the metric to be used for the score can be chosen from several available options. For each of *G* generations (*G* defaults to 100 but can be user-specified), the GP algorithm selects the top 20 pipelines in the current population according to a specific scheme (Deb et al. 2002) that aims at optimizing the average CV score and minimizing the complexity, i.e. the number of steps. These pipelines produce the next generation of the population via transformations that mimic genetics, such as point mutations (random change of one of the pipeline operators) and cross-over of two pipelines (Fig. 3). At every generation, the algorithm updates a *Pareto front* of the pipelines discovered at any point in the GP run, where the Pareto front consists of those pipelines for which there is no other pipeline with both a better average CV score and a smaller complexity. This process iterates for the *G* generations, whereby adding and tuning pipeline operators that improve the average CV score and pruning those that degrade it. At the end, the algorithm selects the pipeline from the Pareto front with the best average CV score as the optimized pipeline and retrains it on the entire data set (i.e., without CV splits). As indicated earlier, it is good practice to evaluate the score of this pipeline on a hold-out testing set. Typically, one approach is to split the original data set into two portions; one (e.g., 75%) to be used as input to TPOT, and the other (e.g., the remaining 25%) as a hold-out set on which to assess the optimized pipeline. Actually, due to the stochasticity inherent to GP, it is also good practice to run TPOT multiple times with different such splits of the original data. Each such run yields a pipeline, and these pipelines can then be explored to get insights into the data. In particular, for each such pipeline, one can rank the features in terms of their importance, and the results can be combined across pipelines.

Since TPOT was first introduced in Olson et al. (2016), several specializations and extensions have been developed which were motivated by biomedical informatics applications. In Sohn et al. (2017), a specialized version of TPOT is introduced that focuses on genetic analysis studies, named TPOT-MDR, where the TPOT search space is constrained to utilize pipelines whose steps use some special operators. The main special operator is an implementation of Multifactor Dimensionality Reduction (MDR), a non-parametric method that combines two or more features to create a single feature that captures their interaction effects (Ritchie et al. 2001) and is, therefore, particularly suited to study epistasis in genetic analyses. Another useful operator employed in TPOT-MDR pipelines is the Expert Knowledge Filter (EKF), which allows feature selection based on statistical or biological filters. This is particularly relevant for applications to data sets comprising a large number of features.

Scalability is an important consideration for the applicability of AutoML to data sets stemming from the omics world. This has inspired two useful extensions of the standard TPOT framework that reduce TPOT computation time (Le et al. 2020). One is the Template which lets the user specify the architecture of pipelines to be searched by TPOT and imposes restrictions on which operator can be chosen at each node. The other is the Feature Set Selector (FSS), which is used in combination with Template, whereby at the very first stage of each pipeline FSS passes only a specific subset of the features onwards. This essentially corresponds to slicing a potentially large original data set into smaller ones allowing TPOT to identify the feature subset that optimizes the *k*-fold CV score. Besides rendering the analyses of large data sets more feasible, the combination of Template and FSS also serves to generate more interpretable models.

Another extension of TPOT which was motivated by applications to biomedical informatics is the ability to adjust for covariates affecting features and/or target (resAdj TPOT). In Manduchi et al. (2020), an approach is presented to do this in a ‘leakage-free’ manner, meaning that the correction is applied in a way that prevents models built on the training part of a CV split from accessing information involving the validation part of the same split. Two other recent TPOT extensions are TPOT-NN, discussed below, and TPOT-cuML which provides a restricted configuration with GPU-accelerated estimators. Among the AutoML pipeline optimizers, TPOT has the most applications to date within the biomedical field, in particular omics applications, so we will discuss these in a separate section below.

### Neural network AutoML

Traditionally, AutoML software tends to build ML pipelines out of relatively simple candidate algorithms. This is due to various reasons, including the relative computational efficiency of training less complex models, portability of simple models to many domains, and sometimes ease of model introspection and interpretability. Despite these reasons, there is increasing interest in using artificial neural networks (ANNs/NNs) within the context of AutoML (Mendoza et al. 2019). NNs are mathematical approximations of biological neural networks, where sets of neurons (simple linear transformations composed with pointwise nonlinearities called *activation functions*) act by accepting input from one or more data points or other neurons, and potentially propagating a signal to subsequent groups of neurons based on whether the inputs pass a threshold specified by the activation function. Neurons are organized into *layers* that are stacked in a serial configuration. *Deep learning* is a branch of ML that uses NNs with many stacked layers; often tens or hundreds of layers. Specific arrangements of neurons result in different NN architectures, each of which has various performance advantages and disadvantages on certain tasks. NNs have exploded in popularity in recent decades, largely due to their ability to approximate any arbitrary function given sufficient size of the network, but also because computers have reached processing speeds that can adequately deal with the very large numbers of tunable parameters these networks contain (billions, in some cases).

The significant flexibility of NN architectures allows for a more general AutoML paradigm to be used when compared to non-NN applications of AutoML. As covered above, most AutoML can be summarized as pipeline optimization, which itself consists of several subtasks, including hyperparameter optimization, feature selection, and others. AutoML software that utilizes NNs can generally fall into two categories: (1) systems that aim to discover a larger NN architecture composed of smaller NN units, an approach known as Neural Architecture Search (NAS) (Elsken et al. 2019); and (2) systems that incorporate simpler, pre-specified NN architectures (such as multilayer perceptrons) as individual operators within a larger ML pipeline. In either of these approaches, NN layers can simultaneously accomplish feature selection (either by ‘dropping out’ or filtering uninformative features, or by explicitly highlighting important features through attention mechanisms (Wang et al. 2017)), classification/regression, dimensionality reduction, and many other tasks that usually need to be explicitly modeled in non-NN systems. A major benefit of NAS is that it can implicitly perform any of these during a single optimization problem. The actual search process of NAS can be accomplished in a number of ways, including via random search, evolutionary methods, Bayesian optimization, and others, which comprise some of the main differences between existing NN-based AutoML systems. The second (non-NAS) approach to NN-based AutoML simply predefines neural networks, possibly with a dynamic number of layers and layer sizes encoded as hyperparameters.

There are several noteworthy AutoML systems that have successfully incorporated ANNs. Here, we provide a brief survey of some of these; we direct readers to (Mendoza et al. 2019) for a detailed technical analysis of some of these NN AutoML systems, as well as several others. AutoGluon—which is discussed more extensively below—is a Python AutoML tool developed by Amazon Web Services that supports NAS, and employs an ensemble stacking and bootstrap aggregation approach to constructing its estimators (Erickson et al. 2020). H2O, also discussed below, is a general-purpose AutoML system that uses a nearly identical approach to build estimators, and also supports NNs (specifically, a simple type of NN known as a multilayer perceptron) through its included deep learning module (Candel and LeDell 2021). H2O’s deep learning capabilities have been used successfully within several biomedical studies, most notably in the context of predicting estrogen receptor status using breast cancer metabolomics data, where the H2O deep learning approach outperformed a wide variety of alternate ML algorithms (Alakwaa et al. 2018). TPOT—which was discussed above—contains a submodule, named TPOT-NN, that provides a flexible framework for defining new NN operators (Romano et al. 2021) that can be incorporated into AutoML pipelines aside non-NN operators supported by base-TPOT. TPOT-NN currently only provides NN implementations of logistic regression and multilayer perceptrons, but the developers have been testing complex neural architectures, including convolutional neural networks (e.g., for image classification) and recurrent neural networks (e.g., for text classification or application to time series data). Furthermore, TPOT-NN provides tools for users to extend the software to make use of any arbitrary NN-based operator that suits their needs.

Google has recently released a new NAS-based tool named Model Search, which is an open-source AutoML tool built on the neural computing library Tensorflow (which is, itself, another open-source software platform written by Google) that combines various strengths of previous AutoML systems. Briefly, Model Search’s strategy for identifying optimal architectures consists of training multiple candidate architectures in parallel, and the results for each candidate are saved in a database. The system then uses a heuristic search algorithm named beam search (Ow and Morton 1988) to compare and rank the results of all candidate models. The best model is then mutated, and the process is repeated for a number of cycles until the system reaches some stopping criterion. Overall, this approach is similar to TPOT/TPOT-NN, where the ranking and mutation strategy of Model Search is analogous to TPOT’s GP optimization approach, but rather than constructing a pipeline from a pool of multi-purpose operators (which may consist of preprocessors, transformers, and a wide variety of ML algorithms), each of the blocks consists of a single neural network “motif” (e.g., a convolutional block, an LSTM block, a ResNet block, or others) that is composed into a larger neural network architecture. In other words, rather than a pipeline, Model Search finds a neural network architecture comprised of smaller blocks of neural network layers, potentially arranged in a branching tree-like configuration.

Several other examples of AutoML applications of NNs exist, but to lesser degrees of popularity. In general, NN applications within AutoML come with several important caveats. Due to the aforementioned large number of parameters, the time required to learn these NNs can be substantially greater than when the AutoML software only considers simpler (i.e., non-NN) estimators. This is sometimes alleviated by performing training on computers with certain hardware that can accelerate the training process (e.g., using CUDA-enabled graphics processing units), but such computing resources are costly and may not be available to all users. Furthermore, the fact that NNs are challenging to introspect and interpret may make these applications unsuitable when it is important to understand why the AutoML system made specific predictions. For example, consider an AutoML analysis of genotype data, where the goal is to predict phenotypic outcomes using SNPs of possibly unknown effects as inputs. If the AutoML system performs exceptionally well, the user may want to understand which SNPs are most predictive of the phenotypic outcome. With simpler ML building blocks (e.g., logistic regression, decision trees, etc.), it can be easy to interpret the contributions of these kinds of input features, but NNs could effectively obscure the effects of individual SNPs due to the complex nonlinear relationships between inputs and outputs of the network. Ultimately, these factors need to be considered on a case-by-case basis, and new research is needed to potentially alleviate these shortcomings.

### Other AutoML approaches

There are several other AutoML approaches, some of which we discuss in this section. We also point the interested readers to MLPlan (Mohr et al. 2018), OBOE (Yang et al. 2019), and Microsoft’s AutoML projects that include FLAML (Wang et al. 2021) and NNI (https://www.microsoft.com/en-us/research/project/neural-network-intelligence/).

#### AutoGluon

Recently, Amazon has open-sourced an AutoML tool named AutoGluon (https://auto.gluon.ai), for Text, Image, and Tabular data. The AutoGluon Tabular (Erickson et al. 2020), which is the most relevant for applications of interest to this readership, does not focus on CASH optimization. Instead, it uses a custom set of base estimators (including RFs and NNs) in a multilayer stacked ensemble scheme. More precisely, in the base layer, these estimators are individually trained. Then, their aggregate predictions are added to the initial features and become the inputs of the next stacked layer, which consists of the same base estimators, and so on until the final step where an ensemble selection like that used in Auto-Sklearn (Caruana et al. 2004) is employed to aggregate the predictions from the last stacked layer in a weighted manner. AutoGluon automatically recognizes the data type for each feature and the type of prediction problem (e.g. regression, classification) and applies model-agnostic pre-processing that transforms the inputs to all estimators followed by model-specific pre-processing that is only applied to the inputs used in a particular estimator. Moreover, the layer-wise training is done in such a way to obtain high-quality data within an allotted time constraint. AutoGluon first estimates the required training time for each estimator in a layer and if this exceeds the remaining time for that layer (based on the allotted time constraint), it skips to the next layer. Base estimators have a predefined order so that the more reliable and less expensive models are trained prior to the less reliable and more expensive ones. Overfitting is mitigated throughout via a careful approach termed ‘repeated *k*-fold ensemble bagging’ which utilizes all the available data for both training and validation, ensuring that the higher layer models are only trained upon lower layer validation predictions. AutoGluon is among the methods evaluated by Seo et al. (2021) for forecasting the walking assistance rehabilitation level of stroke patients based on 82 features in 6 categories (anthropometry, stroke, blood tests, functional assessment, biosignal ward, and disease). We are not aware of omics applications of AutoGluon to date, probably due to its relatively recent release, but it is a promising method for this field.

#### AutoPrognosis

AutoPrognosis is an autoML system tailored to clinical prognosis and able to handle a diversity of clinical data types (including longitudinal and survival data). The approach uses an advanced Bayesian optimization technique to design a prognostic model consisting of a weighted ensemble of ML pipelines (Alaa and van der Schaar 2018a). The system also provides explanations of its predictions in the form of logical association rules linking patients’ features to predicted risk strata. AutoPrognosis was used (Alaa et al. 2019) to develop a cardiovascular disease risk predictor based on ~ 500 features, using a study of 423,604 UK Biobank (Bycroft et al. 2018) participants. Another application was prediction of short-term survival of cystic fibrosis (CF) patients using data from the UK CF registry (Alaa and van der Schaar 2018b).

#### H2O

H2O is a commercial entity providing cloud-based machine learning services. Components of their software are freely available and open-source including software for AutoML. The H2O AutoML software includes a grid search and Bayesian optimization algorithms for hyperparameter tuning and the use of the Super Learner algorithm (van der Laan et al. 2007) which combines multiple machine learning algorithms as an ensemble for prediction (LeDell and Poirier 2020). Super Learner is more extensively discussed below.

H2O, Auto-WEKA, Auto-sklearn, and TPOT are part of an open-source, extensible, and ongoing benchmark for AutoML frameworks publishing online the latest results on the performance of these tools on public datasets (Gijsbers et al. 2019).

#### PennAI

PennAI was designed as an accessible and user-friendly AutoML software package for non-experts (Olson et al. 2018). It features the scikit-learn library for machine learning, a controller for launching jobs, a database for storing machine learning results as a memory of the system, a singular-value decomposition (SVD) algorithm-based recommender system, and a user-friendly interface accessible via web browser. The recommender system analyzes previous machine learning results from the database and automatically launches and runs new analyses. PennAI has been shown to be competitive with Auto-Sklearn and HyperOpt, an automated hyperparameter tuner (Bergstra et al. 2015; Komer et al. 2019). PennAI has been applied to biomedical data in (La Cava et al. 2020). We are not aware of any omics applications of PennAI.

## TPOT omics applications

TPOT has been applied in several omics contexts. Transcriptomics was one of the motivations for the FSS and Template extensions described above and a first application to RNAseq data from individuals with or without major depressive disorder was presented in that paper (Le et al. 2020). In Manduchi et al. (2020), two more extensive transcriptomics applications can be found. The first used a toxicogenomics Affymetrics microarray data set (1693 features and 933 samples) to build models leading to the identification of pathways and genes whose expression is associated with creatinine levels in rat kidney, after utilizing the covariate adjustment extension introduced in that paper to remove confounding effects such as study batch, compound treatment, dose, and sacrifice time. The second used an RNAseq expression data set (4952 features and 1072 samples) to build models leading to the identification of pathways associated with differential expression between schizophrenic and control individuals. Interestingly, the latter yielded known pathways which could not be detected by the more popular gene set enrichment tool GSEA (Subramanian et al. 2005).

**Fig. 4:**
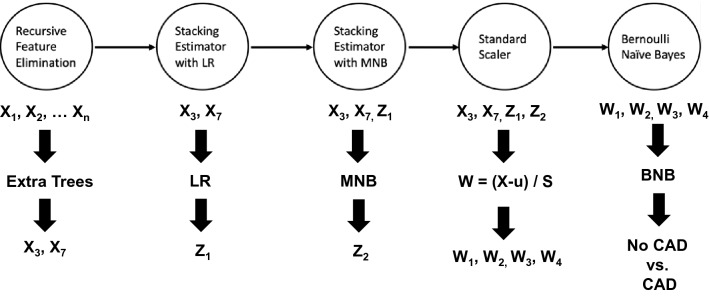
An optimal TPOT pipeline derived from the analysis of metabolomics data (Orlenko et al. 2020). In the first step of the pipeline, an Extra Trees analysis is performed with recursive feature elimination to select a subset of most informative features. These selected features are then analyzed using Logistic Regression (LR) and the output included in the data set as a newly engineered feature. This same process is then repeated using a multinomial naïve Bayes (MNB) algorithm. The selected and engineered features are then scaled by subtracting the mean and dividing by the standard deviation. These newly transformed features are then used to classify subjects as cases with coronary artery disease (CAD) or controls with no CAD using a Bernoulli Naïve Bayes (BNB) classifier

Metabolomics is another area where TPOT has been successfully applied. The first application in this field was presented in Orlenko et al. (2018) to study type 2 diabetes patients with glycemic control exposed to metformin monotherapy as compared to matched healthy controls. In a second metabolomics TPOT application, described in Orlenko et al. (2020), a cohort of 925 patients is analyzed using 73 metabolic and 27 demographic and clinical features with respect to an endpoint of obstructive, non-obstructive, and no Coronary Artery Disease (CAD). Interestingly, in this work, the pipeline discovered by TPOT as having the best classification performance (see Fig. 4) includes the Bernoulli Naïve Bayes classifier. The latter is typically employed in text analyses for spam detection and is rarely considered for biomedical predictive analyses, so it would unlikely be used in a manually configured ML pipeline for this task. The most recent metabolomics application of TPOT utilizes > 500 measurements to build predictive models of early childhood caries, in Heimisdottir et al. (2021).

As for genomics, besides applications of TPOT (Olson et al. 2016) and TPOT-MDR (Sohn et al. 2017) to a data set extracted from a GWAS on prostate cancer aggressiveness, more recently (Manduchi et al. 2021) TPOT and resAdj TPOT were used to analyze a large CAD data set extracted from the UK Biobank, consisting of > 19,000 cases and > 320,000 controls. Functional genomics data from Roadmap Epigenomics (Kundaje et al. 2015), previous results on putative CAD druggable genes (Tragante et al. 2018), and an integrative network resource (https://het.io/) were employed as a biology-based feature filter. A 2-stage TPOT approach yielded the identification of a recurrent signal from a subset of 28 SNPs and feature importance analyses uncovered links between the top SNPs in this subset and genes related to atherosclerotic plaques and myocardial infarction. We will return to this example below where we discuss the challenges presented to AutoML by full-fledged GWAS data sets and potential directions for future development.

Radiomics has also seen an application where TPOT was employed to determine the prognosis of clear cell renal cell carcinoma prior to any invasive therapy on the basis of > 6000 MRI-based features in a cohort of 374 patients (Choi et al. 2021). Aside from the omics area, TPOT is suitable for other types of biomedical predictive tasks, including public health, as recently highlighted in Manduchi and Moore (2021).

## AutoML in genetics; promises, challenges, future directions

Potential applications of AutoML to genetics and genomics are not limited to predictive models for traits based on features derived from genotyping assays. For example, AutoML could be used to possibly improve on methods such as the SNP deleteriousness scorers described earlier. However, the possibility of using AutoML to study phenotype-genotype relationships is probably the most appealing as it offers a means to automatically explore associations that go beyond those investigated with traditional GWAS approaches. It might be ideally suited to genetic analysis in the presence of epistasis, genetic heterogeneity, and gene–environment interactions. These are all genetic phenomena which tend to be non-additive thus requiring machine learning to complement parametric approaches. The challenge for modeling interactions and heterogeneity is knowing which pre-processing and machine learning algorithms are the right ones to use. AutoML has the potential to improve machine learning results by making fewer assumptions about the right methods to use.

There are two main purposes that may drive ML, hence AutoML, applications in the study of phenotype–genotype relationships. In the first and most frequent scenario, the main goal is to identify the genes associated to a given trait. This includes applications where ML is directly used to discover relevant features (e.g., Adams et al. 2020; Manduchi et al. 2021), in which case the emphasis is on feature importance and, more generally, interpretability. It also includes applications where ML is used for post-GWAS prioritization; we are not focusing on these but refer to (Nicholls et al. 2020) for a review. In the second scenario, often motivated by precision medicine applications, the main interest is in the immediate output of the ML i.e., the predictive model itself (Li et al. 2021; Huang et al. 2021), in which case the emphasis is on predictive ability.

A GWAS data set can present many challenges to AutoML analyses. First, the search space is very large, both in terms of number of samples and features (SNPs). Second, the signal is usually weak as common variants typically have small effect sizes. In fact, one of the reasons GWAS comprise very large numbers of samples (often combining several cohorts under the umbrella of consortia) is to provide enough power to detect such signals. Third, a trait may present genetic heterogeneity, meaning that different variants may be responsible for that trait in different individuals, and this increases the difficulty in detecting the signals. Finally, for binary traits, often there is a large imbalance between the number of cases and controls. This is, for example, true for data collected as part of national biobanks, like the UK Biobank (https://www.ukbiobank.ac.uk/) or All of Us (https://allofus.nih.gov/). To date, applications of AutoML to GWAS data are very few and we have indicated those that we know of above. Computational feasibility is certainly a major hurdle. Reducing the number of SNPs is generally the first step and is also needed to handle the *p* >  > *n* issue. Systems like Auto-sklearn and TPOT comprise various (computationally based) feature selectors and transformers among the operators of the explored pipelines, and TPOT Template allows to specify that one of these operators should be the first step of any explored pipeline. In these pipelines, the dimensionality of what is passed to subsequent classifier/regressor operators is, therefore, reduced. Moreover, the entire pipeline, including the feature selector/transformer step, is assessed via CV. However, with GWAS data containing millions of SNPs (the large majority of which have values in {0, 1, 2}), in current AutoML systems, it is still typically necessary to filter SNPs prior to running the program. Employing computationally based filters to this end may not be ideal, also because this process would not be leakage free. Filters based on domain-specific knowledge are independent of the training data and provide an attractive approach, especially when the emphasis is on interpretability. When using this type of filter, the goal is no longer to look for all possible signals of interest but to focus on a promising subset. SNP filters can be based on biological knowledge, e.g. restricting the analyses to SNPs in pathways that are known to be relevant to the trait, as is done in Olson et al. (2016) and Sohn et al. (2017). Another approach, as in Manduchi et al. (2021), is to integrate various resources, including functional genomics data from tissues that are relevant to the trait as well as the computational scorers for variant deleteriousness described above. But there are difficulties that come along with this. For example, for some traits, the culprit tissue(s) may not be fully known or the functional genomics data for known culprit tissues may not be available. Even when the tissues are known and the data are available, the reliability of the derived regulatory regions (e.g., enhancers) or SNP functional scores are tied to the computational methods used and it is possible that the trait-relevant SNPs are erroneously removed from the search space. In addition, filtering of SNPs as just described, typically still yields more features than an AutoML system can handle. To overcome this, the FSS option in TPOT can be valuable. For example, in Manduchi et al. (2021), SNP sets are created based on pairs of connected genes in the Hetionet integrative network and each searched pipeline starts with the step of selecting one of these sets. An additional advantage of using the FSS is that it is also a way of facilitating interpretability. More efficient handling in AutoML of feature selection and feature transformation approaches in general, represents an avenue for future developments to improve AutoML applicability to genomics. Including selectors that are not based on main effects, like the RELIEFF based selectors (Kononenko et al. 1997) used in TPOT-MDR, is desirable to better explore potential epistatic effects. As for feature transformations, efficient incorporation within the AutoML of feature fusion approaches such as, for example, that discussed in Venugopalan et al. (2021), is another interesting area for development.

While filtering and engineering SNPs to reduce dimensionality is an important area of investigation, it is important to note that there are machine learning algorithms such as deep learning neural networks and gradient boosting methods which can scale to genome-wide genetic and genomics data. These are especially attractive when the emphasis is on predictive ability. For example, deep learning has been applied to the analysis of GWAS data from studies of Alzheimer’s disease (Li et al. 2021). One of the challenges of deep learning is that there are a number of hyperparameters which need to be tuned. The Auto-Net methods was designed to automate the construction and hyperparameter tuning of deep learning models (Mendoza et al. 2019). This approach is synergistic with Auto-Sklearn and, as discussed above, opens the door to AutoML using neural networks. The scaling of deep learning to GWAS and other genome-wide studies with AutoML methods such as Auto-Net needs to be evaluated. Another approach to address scalability is to take advantage of an increasing number of cloud-based commercial AutoML solutions. These are quickly becoming mature and are integrated with cloud-based high-performance computing which makes scaling easy. The advantages and disadvantages of these solutions for AutoML analysis of genome-wide data should also be explored.

Even after filtering SNPs and grouping them in feature sets, the number of samples from GWAS is typically too large for current AutoML capabilities. In this case though, rather than filtering subjects, one could instead run the AutoML several times using different subsets of the subjects. For example, in Manduchi et al. (2021), TPOT is run multiple times, each time using all available cases and a subset of the controls of the same size as the cases, which also has the effect of balancing the input of each run. Other schemes are possible and could involve imbalance-aware approaches such as that described in Schubach et al. (2017), leveraging packages like the Python imbalanced-learn (https://imbalanced-learn.org/stable/). Another interesting avenue to explore, for better tailoring of AutoML to genomics in view of large sample sizes, is incorporation of approaches like FABOLAS (Klein et al. 2017), a Bayesian optimization method that evaluates models on subsets of the data to learn good hyperparameter settings, aiming to assess configurations that yield, per time spent, the most information about the globally best hyperparameters for the full dataset.

We note that, even with SNP filtering and multiple runs on subsets of the samples, the computational resource requirements can be demanding and having the availability of a computer cluster for parallel runs of the AutoML is highly desirable. As more progress is made to leverage GPU for AutoML to improve scalability and GPU cost decrease, we expect analysis of GWAS data using these systems to become more common.

When a given AutoML is run multiple times, either for the reasons indicated in the previous example or simply due to inherent stochasticity (like in TPOT), multiple optimal pipelines are generated. When the emphasis is on interpretability, feature importance can be computed for each such pipeline and the results combined to get insights. But it is also possible to combine these pipelines with ensemble methods, either simple approaches such as voting or more sophisticated approaches such as Super Learner, so to obtain a single model at the end, which may outperform the individual models upon which it is built. (This is useful both when the emphasis is on interpretability and predictive ability.) Super Learner is an ensemble-based algorithm that is particularly attractive for AutoML for two primary reasons. First, it is compatible with any arbitrary machine learning algorithm to comprise its individual components. Other ensemble methods often do not have this flexibility—for example, gradient tree boosting algorithms rely on individual learning functions with differentiable losses (many sophisticated AutoML approaches generate learners that might not have computable gradients). Furthermore, there are often technical limitations related to the complexity of the individual candidate learners, such as AdaBoost relying on simple and quickly trained candidate learners that perform classification or regression only slightly better than average. Second, Super Learner is analytically known to (asymptotically) produce an ensemble learner that is as accurate as the best possible prediction algorithm. Briefly, Super Learner trains *V* arbitrary candidate learners, each on a collection of data with a different validation block of samples held out from the learning process. Each candidate learner is then used to predict outcomes on samples from their individual validation blocks, and the overall body of predictions is then used to train a regression model that assigns relative weights to the predictions of each candidate algorithm. Super Learner—out of the context of AutoML—has been successfully used to a substantial degree in biomedical data science (Sinisi et al. 2007; van der Laan et al. 2007; Golmakani and Polley 2020). TPOT is currently in the process of developing an extension that combines individual AutoML pipelines into a Super Learner.

One of the important limitations of many machine learning methods and applications is that only one objective (e.g., classifier accuracy or area under the ROC curve) is used to evaluate the quality of the model. This may work well for some problems. However, there may be additional objectives of importance for problems in genetic and genomics. For example, a machine learning model designed to identify genes representing new drug targets might care about whether there is evidence that the protein products are druggable. A model with no druggable genes might predict a phenotype with a high accuracy but might not be useful or interesting from a pharmacology perspective. Fortunately, there are methods for assessing the quality of a model using multiple objectives. An example of a multi-objective algorithm is Pareto optimization which ranks models according to two or more objectives. Models are referred to as Pareto optimal if one objective cannot be improved by selecting another model without being worse for one or more other objectives. The TPOT AutoML approach uses a type of Pareto optimization called non-dominated sorting (Deb et al. 2000) and by default selects Pareto optimal models using accuracy and pipeline complexity. These objectives could be modified to include biological criteria such as druggability. It will be important to evaluate whether biology-based objectives improve AutoML for genetic and genomics problems beyond standard metrics such as accuracy.

Automated machine learning shows tremendous promise for the genetic analysis of complex traits. Particularly important is the ability of AutoML to bring machine learning to non-experts by taking much of the guesswork out of selecting algorithms and hyperparameters. High-throughput genetic data have specific characteristics that distinguish them not only from the tabular data which are more typically used in ML and AutoML, but also from other types of omics data. They are much larger scale both in terms of number of features and samples, they can be highly imbalanced (e.g., when derived from biobanks), imputation of missing values requires sophisticated and time consuming methods (Shi et al. 2018), it is typically important to identify relevant mechanisms (e.g., genes) hence interpretability is often a requirement. We note that the latter is further complicated by the fact that, even when relevant SNPs are identified, subsequent identification of the genes affected by these SNPs is non-trivial, since the affected genes are not necessarily those closest to the SNP in the linear genome and typically integration of additional data sources (other types of functional genomics data) is necessary to further elucidate mechanisms. Thus, several areas need to be developed further before the full potential of AutoML methods can be realized in the genetics domain. First, many of these approaches are computationally intensive, because they are iterating over many different algorithms. Additional work is needed to scale AutoML to genome-wide data, for example. Second, genetic analysis has the potential to be informed by functional genomics data. It will be important to develop powerful methods which allow AutoML to take advantage of these data for model building and search. Third, interpretation is always an important challenge for any ML result. AutoML has the potential to develop complex ML pipelines. The combination of multiple different ML algorithms in a pipeline can make interpretation more challenging. Finally, enabling geneticists with no ML experience to use AutoML will be key to maximizing the value of omics data we have collected for the study of complex traits. An emphasis on accessible and user-friendly software will be essential. We look forward to a time when anyone who wants to use ML for genetic analysis can do so with ease of running a *t* test.

## Data Availability

Not applicable.
